# Symptoms of anxiety and depression in patients with primary hyperhidrosis and its association with the result of clinical treatment with oxybutynin

**DOI:** 10.6061/clinics/2021/e2892

**Published:** 2021-06-23

**Authors:** Débora Yumi Ferreira Kamikava, Nelson Wolosker, Marcelo Fiorelli Alexandrino da Silva, José Ribas Milanez de Campos, Pedro Puech-Leão

**Affiliations:** I Divisao de Psicologia, Instituto Central, Hospital das Clinicas HCFMUSP, Faculdade de Medicina, Universidade de Sao Paulo, Sao Paulo, SP, BR; II Disciplina de Cirurgia Vascular e Endovascular, Hospital das Clinicas HCFMUSP, Faculdade de Medicina, Universidade de Sao Paulo, Sao Paulo, SP, BR; III Hospital Israelita Albert Einstein, Sao Paulo, SP, BR; IV Disciplina de Cirurgia Toracica, Hospital das Clinicas HCFMUSP, Faculdade de Medicina, Universidade de Sao Paulo, Sao Paulo, SP, BR

**Keywords:** Hyperhidrosis, Anxiety, Depression, Oxybutynin

## Abstract

**OBJECTIVES:**

Studies have identified correlations between the psychological characteristics of individuals with primary hyperhidrosis (HH), the degree of sweating, and the quality of life (QoL). This study aimed to evaluate the prevalence of anxiety and depression symptoms in patients with HH before and after oxybutynin treatment.

**METHODS:**

Data were collected from 81 patients. Palmar or axillary HH was the most frequent complaint (84.0%). All patients were evaluated before the medication was prescribed and after five weeks of treatment. The Beck Depression Inventory and Beck Anxiety Inventory were used to evaluate depression and anxiety.

**RESULTS:**

Improvement in HH occurred in 58 patients (71.6%), but there was no improvement in 23 patients (28.4%). The QoL before treatment in all patients was either “poor” or “very poor.” Patients who experienced improvement in sweating rates also experienced a greater improvement in QoL than patients who did not experience improvement in sweating at the main site (87.9% *vs.* 34.7%) (*p*<0.001). A total of 19.7% of patients showed an improvement in their level of depression, and a total of 46.9% of patients exhibited improvements in their level of anxiety. A significant correlation was observed between sweating and anxiety (*p*=0.015).

**CONCLUSION:**

Patients with HH who experienced improvements in sweating immediately after treatment with oxybutynin exhibited small improvements in their levels of depression and significant improvements in their levels of anxiety and QoL.

## INTRODUCTION

Hyperhidrosis (HH) is a somatic disorder that involves excessive perspiration beyond physiological needs in one or more specific regions of the body due to hyperfunctioning sweat glands. It is often triggered by emotional situations with no obvious etiology (1-3).

HH affects approximately 2.8% of the population (4), particularly adolescents and young adults. It is associated with family history (5), and it can affect the hands (the most frequently affected region) and the axillary, facial, and plantar regions (6). All clinical presentations lead to social, professional, and affective problems and may even lead to social phobia (7).

Currently, there are surgical and clinical treatments for HH that differ in terms of efficiency, effectiveness, duration of effect, side effects, and scientific validation. Laparoscopic thoracic sympathectomy is the most effective surgical treatment for presenting functional and long-lasting results, but its main complication is compensatory sweating. Clinical treatments can be topical or systemic via antiperspirants, botulinum toxin, or other medications (1,7).

The treatment for all HH forms in our institution consisted of initial treatment with oxybutynin for five weeks. When the result is satisfactory, the medication is continued indefinitely; however, when the result is unsatisfactory, sympathectomy is indicated if the patient is in good clinical condition (8,9).

Studies have identified correlations between the psychological characteristics of individuals with HH, the degree of sweating, and the quality of life (QoL). Braganca et al. (10) observed a higher prevalence of anxiety in patients with HH than in the general population. Depression symptoms were either less prevalent in patients with HH or were similar to those in the general population. However, depression symptoms are associated with anxiety in patients with HH. Other studies have revealed that emotional issues such as stress and anxiety are aggravating factors of HH and, consequently, interfere with the QoL (11-13).

Although several studies have evaluated the variation in patients’ QoL with HH before and after treatment, few studies have evaluated the symptoms of anxiety and depression in patients with HH (10,14-20). To our knowledge, no study has analyzed the changes in the symptoms of anxiety and depression after the clinical treatment of HH with oxybutynin.

The aim of this study was to evaluate the prevalence of symptoms of anxiety and depression in patients with HH before and after oxybutynin treatment by analyzing the relationship between the variations in these symptoms and the effectiveness of the drug in improving HH.

## MATERIALS AND METHODS

This was a prospective, single-center study. From June 2017 to March 2018, data were collected from 81 consecutive patients with HH who were treated with oxybutynin. The same protocol was used for all patients. The only contraindications for the use of oxybutynin include hypersensitivity to medications and presence of angle-closure glaucoma. None of the patients in this study had these characteristics.

Table 1 shows the patient demographics. Most of the patients were women, eutrophic (body mass index [BMI] of 23), and in the third decade of life. Palmar or axillary HH was the most frequent complaint (84.0%), as shown in Table 2.

Oxybutynin was prescribed for five weeks at progressively increasing doses throughout the treatment period. On their first visit, the patients were given 2.5 mg of oxybutynin to be taken once a day in the evening. They were instructed to increase the dose to 2.5 mg twice a day during the second week and to 5 mg twice a day from the 15^th^ day to the end of the 35th day when the last visit was scheduled.

All patients were evaluated on two occasions: before the medication was prescribed and after five weeks of treatment. At the first visit, participants were asked to complete three validated questionnaires. First, sweat at each site was quantified using the Hyperhidrosis Disease Severity Scale (HDSS), and the negative impact of HH on QLV was analyzed according to the protocol originally developed by Amir et al. (21), which was subsequently translated to Portuguese (22). Second, the Beck Depression Inventory (BDI) and Beck Anxiety Inventory (BAI) were used to evaluate depression and anxiety.

The HDSS consists of a direct and straightforward question with four available answers related to the patient’s tolerance to HH symptoms and the negative implications of excessive sweating in their daily activities.

The Beck Scales were developed by Aaron Beck and his colleagues in 1961 and are composed of four scalar measures. The BDI and BAI are self-report instruments with descriptive items of attitudes and symptoms that aim to measure the intensities of depression and anxiety symptoms. These instruments are not indicated for the diagnosis of specific conditions (42).

BDI is indicated for subjects between the ages of 17 and 80 years and consists of 21 items related to sadness, pessimism, feeling of failure, dissatisfaction, guilt, punishment, self-aversion, self-accusations, suicidal ideas, crying irritability, social withdrawal, indecision, change in self-image, difficulty working, insomnia, fatigue, loss of appetite, weight loss, somatic concerns, and loss of libido. The BAI is also indicated for subjects between the ages of 17 and 80 years and consists of 21 items associated with the usual symptoms of anxiety in each patient with reference to themselves; it describes the level of severity of each symptom.

The negative impact of HH on QoL before treatment was classified into five different levels and were calculated as the summed total score from the protocol (range, 20-100). Higher levels indicate poorer QoL. When the total scores were >84, 68-83, 52-67, 36-51, and 20-35, the QoL levels were considered very poor, poor, good, very good, and excellent, respectively.

Depression was categorized into four different levels: 0-11 points, minimum; 12-19, mild; 20-35, moderate; and 36-63, severe.

Anxiety was also categorized into four levels: 0-10, minimum; 11-19, mild; 20-30, moderate; 31-63, severe.

After 5 weeks of treatment, the patients were asked to complete three questionnaires. For the level of symptoms and QoL, the questions were the same as those used during pharmacological pretreatment; however, the patients were asked to respond to each question by using “better,” “same,” or “worse than before the treatment” as answers. The results of oxybutynin treatment were analyzed by considering the primary location. When there was no variation in the level of sweating, we considered the improvement to be null. When there was a variation in the scale from a higher (more intense) to a lower (less intense) level, we concluded that there was an improvement in HH. Based on the degree of improvement in sweating, which was evaluated using the HDSS questionnaire, the patients were divided into the improvement group and stable group.

The improvement in QoL after treatment was classified into five levels. When the total scores were >84, 68-83, 52-67, 36-51, and 20-35, the QoL levels were considered to be much worse, a little worse, the same, slightly better, and much better, respectively.

The following parameters were analyzed and compared between the two groups: negative impact of HH on QoL before treatment, improvement in QoL after treatment, changes in the levels of anxiety and depression, and impact of improving HH on the levels of anxiety and depression.

We used two methods to analyze the changes in the levels of depression. First, we studied the prevalence of the different levels of anxiety and depression in the group before and after the treatment. Second, we studied the variation in the levels of anxiety and depression in each individual before and after treatment. If there was stability, we considered the variation to be null (0). The values were negative and positive if the levels worsened and if there was an improvement, respectively. If the levels of anxiety or depression in an individual increased or decreased by one level, two levels, and three levels, we assigned a score of one, two, and three, respectively.

### Statistical analysis

The qualitative characteristics of the patients were described using absolute and relative frequencies, and the quantitative characteristics of the patients were described using summary measures (mean, standard deviation, median, minimum, and maximum). The values and levels of anxiety and depression were compared before and after drug treatment by using paired Wilcoxon tests, and the variation in the depression levels between groups before and after treatment were compared using the McNemar test.

We also used the Spearman test to assess the relationship between variations in either anxiety or depression with the evolution of QoL and with the evolution of sweating.

The significance level for all statistical tests was 5%.

### Ethics

The institutional ethics committee approved this study, and all patients signed an informed consent document prior to inclusion. The protocol used for the patients was in accordance with the hospital’s ethical standards set by the Ethics Committee for Analysis of Research Projects on Human Experimentation.

## RESULTS

An improvement in HH, which is based on the degree of improvement in sweating and was evaluated using the HDSS questionnaire, occurred in 58 patients (71.6%), whereas 23 patients (28.4%) did not experience any improvements. None of the patients experienced a worsening of their clinical condition.

As an example of a change in the levels of anxiety and depression after the treatment of HH, we have a 38-year-old female patient who was diagnosed with palmar and plantar HH. In the pretreatment evaluation, she scored 33 (severe) for anxiety and 19 (mild) for depression. After 5 weeks of treatment with oxybutynin and significant improvements in sweating degrees, her score decreased to 7 (minimum) for anxiety and 10 (minimum) for depression. This indicates a variation of three levels for anxiety and one level for depression. In the initial interview, the patient described intense discomfort and suffering related to MH symptoms and indicated to having already missed a job opportunity; she worked as an event decorator, and HH interfered with her ability to coordinate events and handle decorative materials, which can be damaged by sweat. After five weeks of treatment, she described total satisfaction with the medication’s effect in reducing sweating and anxious behavior, and this led to improvements in her QoL.

The QoL before treatment in all patients was either “poor” or “very poor.” There was a significant predominance of very poor QoL in the improvement group, as observed in Table 3 (70.1%, *p*=0.42).

Table 4 shows the improvement in QoL. None of the patients complained of deterioration in their QoL after treatment. Patients who experienced an improvement in sweating rates also experienced greater improvement in QoL than patients who did not experience improvement in sweating at the main site (87.9% *vs.* 34.7%, *p*<0.001).

The only adverse effect associated with oxybutynin treatment was dry mouth, which was observed in 50 cases (61.7%). None of the patients required treatment discontinuation.

There was a greater concentration of minimum intensity of depression after treatment (75.3% *vs.* 66.7%, *p*=0.047) (Table 5), and there was an improvement in the level of depression in 19.7% of patients. There was also a greater concentration of milder anxiety intensity after treatment (74.1% *vs.* 42%) (Table 5), and 46.9% of patients exhibited improvements in anxiety intensity (Table 5).

Table 6 shows the variations in the intensities of anxiety and depression in the whole group after treatment. There was a slight improvement in depression and a significant improvement in anxiety.

The Spearman correlation test did not show a relationship between variations in anxiety (*p*=0.083) or depression (*p*=0.259) and the evolution of QoL. There was also no relationship between the variation in depression and the evolution of sweating (*p*=0.183). However, a significant correlation was observed between sweating and anxiety (*p*=0.015).

## DISCUSSION

Sweating is a physiological mechanism of human thermoregulation. Sweat gland hyperfunction or HH is a very common disease that affects young people during their maturation phase (23). Our patients were in the third decade of life, and more than 80% of patients experienced palmar or axillary HH. Female sex was predominant, and this was a characteristic that was also observed in other studies probably because women have greater aesthetic social concerns and seek help more frequently than men (10,19,24).

To objectively assess HH evolution, we used the widely used HDSS questionnaire (12). Sweating can also be evaluated using equipment that measures the evaporation of sweat from the skin or by using sweat meters; however, we believe that these machines do not reflect the daily suffering of an individual. A patient’s impression is more important (25,26).

Until 1995, surgical cervicothoracic sympathectomy was the only therapeutic alternative for the treatment of HH. Although this therapy could decrease sweating, it causes Horner syndrome, which is a frequent side effect; this is not acceptable (27). In the second half of the 1990s, institutions began performing video-assisted sympathectomy, thus almost completely abolishing the risk of Horner’s syndrome. This is why the number of video-assisted sympathectomy procedures has increased. However, this procedure leads to compensatory HH, a side effect that is also very embarrassing to patients (28). Starting from the end of the 2000s, several scientific studies have been conducted, and this has resulted in the use of oxybutynin. In our institution, oxybutynin is used as a first-line treatment. Oxybutynin is indicated for both sexes (13) unlike surgery, and it can be used to treat patients of all age groups (11,29-31) and obese and nonobese patients (32). It yields good results (improves sweating in more than 70% of patients) in the short and long term (33-35). The only contraindications are hypersensitivity to medication and presence of angle-closure glaucoma.

The results obtained in this study are congruent with those reported in the literature. We observed improvements in sweating in 71.6% of patients, similar to those reported in previous studies (22). Among the patients who did not experience improvements, 28.4% were referred to other therapeutic alternatives, particularly video-assisted thoracic sympathectomy, whenever their clinical conditions were good.

QoL questionnaires are important tools for evaluating treatment results in medicine (25). The use of questionnaires in clinical research enables the collection of high-quality data for the diagnostic and follow-up evaluations of patients (42). The effect of sweating on QoL has also been well studied. Questionnaires are easy to apply and, in our opinion, very important. We routinely use questionnaires as part of the clinical evaluation of patients (9). Depending on the location and intensity, HH can cause embarrassment; discomfort; and even serious social, professional, and psychological problems, thus affecting daily activities and professional careers (37). All patients in our series had poor or very poor QoL before treatment. This was expected because patients with good QoL do not usually seek treatment.

A reduction in sweat production is the main objective of the treatment. When achieved, it leads to an improvement in QoL by reducing psychological limitations. This occurs despite the side effects, which in most cases are not considered significant by patients (36). In the current study, 85% of those who experienced improved sweating at the main site had a significantly better QoL than patients who did not experience improvements in sweating. In addition to the QoL, which has been studied for more than 15 years, HH can also cause psychological and relationship disturbances (19,38). Considering that HH appears predominantly during childhood and adolescence, it can significantly affect mental health because these age groups have a certain predisposition to the development of some types of psychopathology (39).

Depression and anxiety are highly prevalent disabling disorders in the general population (with rates between 12.2% and 48.6%) and are associated with a low QoL (40). Such a variation may be due to the type of population and the diagnostic instruments used. Few medical conditions have a greater global epidemiological impact than depression. This is a complex, multifactorial, chronic, recurrent, and heterogeneous disorder that is often associated with poor general medical conditions and leads to negative consequences (43).

Previous studies suggest that the prevalence of depression in individuals with HH is similar to that in the general population; however, the anxiety level in patients with HH is higher (10). Therefore, psychosocial aspects are important in the management of these patients.

To the best of our knowledge, this study is the first study to investigate whether a reduction in excessive sweating by oxybutynin would change the levels of depression and anxiety. Weber et al. (19) studied a small sample of 31 patients with primary HH and noted that anxiety and depression improved after treatment with botulinum toxin.

In this study, 12.4% of patients had a moderate or high level of depression before treatment. After treatment, this proportion of patients decreased to 8.6%. This improvement was statistically significant but was relatively small. However, we did not find a statistical relationship when we analyzed the relation of improvement in sweating and the improvement in depression.

There was a significant improvement in the anxiety symptoms. Before treatment, 30.8% of patients had moderate or high symptoms, and this proportion decreased to 13.5% after treatment with oxybutynin. This improvement is clearly related to the improvement in sweating.

Anxiety and HH may share the same etiopathogenic pathways and may be related to autonomic overexcitation as a form of intermittent dysautonomia. These patients suffer both from the discomfort caused by excessive sweating and the fear of being seen and negatively judged by third parties, which can lead to avoidance, social isolation behaviors, or even hypervigilance (44).

The limitations of this study include the small number of patients and the relatively short study period of five weeks, which did not allow us to study the level of psychological symptoms over the long term. Involving a larger number of patients would have yielded a more accurate result for the efficiency of this medication and would have allowed us to distinguish between patients with axillary HH and those with palmar HH. Further studies involving a large group of patients treated with oxybutynin for a longer period would reveal how they evolve over time.

## CONCLUSIONS

Patients with primary HH who experienced improvements in sweating immediately after treatment with oxybutynin exhibited small improvements in the levels of depression and significant improvements in the levels of anxiety and QoL. An improvement in anxiety was directly associated with an improvement in sweating.

These results emphasize the importance of treating HH immediately after it is diagnosed to prevent patients from experiencing possible emotional consequences due to HH.

## AUTHOR CONTRIBUTIONS

Kamikava DYF was responsible for the manuscript conception, research, development and accountability. Wolosker N performed the critical revision and approval of the final version of the manuscript. Silva MAF was involved in the manuscript conception and critical review. Campos JRM contributed to the study as a researcher. Puech-Leão P was responsible for the manuscript conception, critical review, and approval of the final version of the manuscript.

## Figures and Tables

**Table 1:** Patient demographics.

Male	24 (29.6%)
Female	57 (70.4%)
Age (range in years)	18-66
Age (average±standard deviation, years)	28.7±10.3
Age (median, years)	27
BMI (average±standard deviation)	24.3±4.3
BMI (median)	23,6
BMI (range)	17.3-39.1

**Table 2:** Primary site of hyperhidrosis as the chief complaint.

Site	n (%)
Palmar	39 (48.2)
Axillar	29 (35.8)
Facial	6 (7.4)
Plantar	5 (6.2)
Back and Buttocks	2 (2.4)
Total	81 (100)

**Table 3:** Impact of hyperhidrosis on QoL before the treatment.

QoL before treatment (Amir questionnaire)	Result of treatment on HH	
	Stable (HDSS questionnaire) n (%)	Improvement (HDSS questionnaire) n (%)
Excellent	0	0
Very good	0	0
Good	0	0
Poor	8 (34.7%)	15 (25.8%)
Very poor	15 (63.3%)	43 (74.2%)
Total	23 (100)	58 (100)

*p*=0.422.

**Table 4:** Changes in the QoL after treatment.

Change in the QoL (Amir questionnaire)	Result of treatment on HH	
	Stable (HDSS questionnaire) n (%)	Improvement (HDSS questionnaire) n (%)
Much better	0	22 (37.9)
Slightly better	8 (34.7)	29 (50)
The same	15 (63.3)	7 (12.1)
Slightly worse	0	0
Much worse	0	0
Total	23 (100)	58 (100)

*p*<0.001.

**Table 5:** Concentration of anxiety and depression intensities in the patients before and after treatment with oxybutynin (n=81).

Intensity	Anxiety		Depression	
	Before n (%)	After n (%)	Before n (%)	After n (%)
Minimum	34 (42.0)	60 (74.0)	54 (66.7)	61 (75.3)
Mild	22 (27.2)	15 (18.5)	17 (21.0)	13 (16.0)
Moderate	18 (22.2)	5 (6.2)	8 (9.9)	6 (7.4)
Severe	7 (8.6)	1 (1.2)	2 (2.5)	1 (1.2)

*p*=0.047, McNemar test for depression intensity.

*p*<0.001, McNemar test for anxiety intensity.

**Table 6:** Variation in the intensities of depression and anxiety with oxybutynin treatment (n=81).

Variation	Depression n (%)	Anxiety n (%)
-1	2 (2.5)	2 (2.5)
0	63 (77.8)	41 (50.6)
1	14 (17.3)	24 (29.6)
2	1 (1.2)	12 (14.8)
3	1 (1.2)	2 (2.5)
